# Frequency of pneumothorax and haemothorax after primary open versus closed implantation strategies for insertion of a totally implantable venous access port in oncological patients: study protocol for a randomised controlled trial

**DOI:** 10.1186/s13063-015-0643-z

**Published:** 2015-03-31

**Authors:** Felix J Hüttner, Tom Bruckner, Ingo Alldinger, Roland Hennes, Alexis Ulrich, Markus W Büchler, Markus K Diener, Phillip Knebel

**Affiliations:** Department of General, Visceral and Transplantation Surgery, University of Heidelberg, Im Neuenheimer Feld 110, 69120 Heidelberg, Germany; Institute of Medical Biometry and Informatics, University of Heidelberg, Im Neuenheimer Feld 305, 69120 Heidelberg, Germany; Study Centre of the German Surgical Society (SDGC), University of Heidelberg, Im Neuenheimer Feld 110, 69120 Heidelberg, Germany

**Keywords:** Venous access ports, Implantation strategies, Pneumothorax, Haemothorax

## Abstract

**Background:**

The insertion of central venous access devices, such as totally implantable venous access ports (TIVAPs), is routine in patients who need a safe and permanent venous access. The number of port implantations is increasing due to the development of innovative adjuvant and neo-adjuvant therapies. Currently, two different strategies are being routinely used: surgical cut-down of the cephalic vein (vena section) and direct puncture of the subclavian vein. The aim of this trial is to identify the strategy for the implantation of TIVAPs with the lowest risk of pneumothorax and haemothorax.

**Methods/Design:**

The PORTAS-3 trial is designed as a multicentre, randomised controlled trial to compare two implantation strategies. A total of 1,154 patients will be randomised after giving written informed consent. Patients must be over 18 years of age and scheduled for primary implantation of a TIVAP on the designated side. The primary endpoint will be the frequency of pneumothorax and haemothorax after insertion of a TIVAP by one of two different strategies. The experimental intervention is as follows: open strategy, defined as surgical cut-down of the cephalic vein, supported by a rescue technique if necessary, and in the case of failure, direct puncture of the subclavian vein. The control intervention is as follows: direct puncture of the subclavian vein using the Seldinger technique guided by sonography, fluoroscopy or landmark technique. The trial duration is approximately 36 months, with a recruitment period of 18 months and a follow-up period of 30 days.

**Discussion:**

The PORTAS-3 trial will compare two different TIVAP implantation strategies with regard to their individual risk of postoperative pneumothorax and haemothorax. Since TIVAP implantation is one of the most common procedures in general surgery, the results will be of interest for a large community of surgeons as well as oncologists and general practitioners. The pragmatic trial design ensures that the results will be generalizable to a wide range of patients.

**Trial registration:**

The trial protocol was registered on 28 August 2014 with the German Clinical Trials Register (DRKS00004900). The World Health Organization’s Universal Trial Number is U1111-1142-4420.

**Electronic supplementary material:**

The online version of this article (doi:10.1186/s13063-015-0643-z) contains supplementary material, which is available to authorized users.

## Background

Totally implantable venous access port (TIVAP) implantation was first introduced in the early 1980s by Niederhuber *et al*. [1]. Following further refinement, the technique has been adopted worldwide to create a safe and permanent venous access for chemotherapy (CHT) and/or parenteral nutrition. TIVAPs last longer, have lower infection rates and are more suitable for active patients than external central venous catheters (such as the Hickman´s catheter) [2]. The number of TIVAP insertions is constantly increasing. In Germany, 480,176 new cases of oncological diseases were diagnosed in 2010. In 2008, 80,034 TIVAP implantations were performed on in-patients in German hospitals, and it is estimated that more than 101,000 TIVAP implantations were performed in an out-patient setting in 2012 [3].

The techniques most often used for insertion of TIVAPs are surgical cut-down of the cephalic vein ((SCD) open strategy), accomplished predominantly by surgeons, and puncture of the subclavian vein ((PSV) closed strategy), performed by surgeons or interventional radiologists. The median primary success rates given in various retrospective studies and a small number of single-centre, randomised controlled trials range from 71 to 94% for SCD, and from 90 to 99% for PSV [2,4-13]. Common complications, such as kinking or dislocation of the catheter, subcutaneous haematoma, wound infection and nerve palsy, are observed with both techniques; however, specific risks are associated only with PSV, including the ‘pinch-off’ phenomenon, pneumothorax and haemothorax, which in most cases requires further treatment and often admission to hospital [14-16]. In the literature, the rate of pneumothorax and haemothorax after PSV is reported to be about 3%, ranging up to 6% in prospective studies [14-18]. In contrast, if SCD is successful there is no risk for pneumothorax or haemothorax; however, in some cases implantation via SCD is not possible, so PSV is required to implant the TIVAP. If SCD fails, a rescue technique (RT) can be executed using a guide wire, vein dilator and peel-away sheath through the cephalic vein to overcome narrow vessels or obstructions, thus avoiding PSV and its subsequent risks. Although TIVAP implantation is one of the top 50 (surgical) interventions in Germany, no multicentre trial comparing these two strategies has been performed.

## Methods/Design

### Aim of the trial

The PORTAS-3 trial, a multicenter, randomized, controlled trial comparing complication rates of open and closed implantation strategies for insertion of a totally implantable venous access port (TIVAP) in oncological patients, is a pragmatic trial that aims at comparing the open strategy (SCD and if necessary RT; if both fail, PSV) to the closed strategy (PSV) with regard to the frequency of pneumothorax and haemothorax.

### Sample size

**Figure 1:**
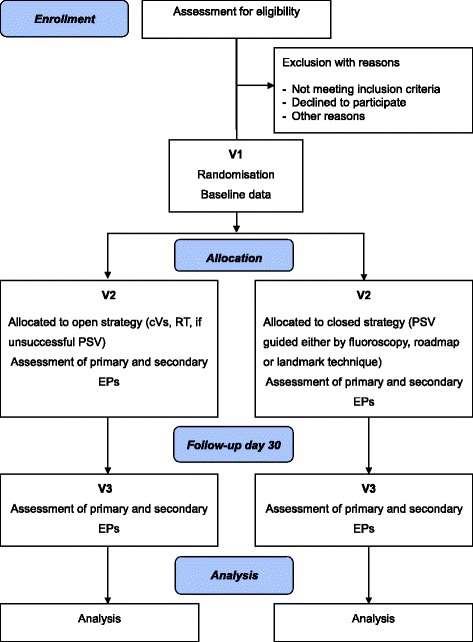
**CONSORT flow chart.** cVS – classical vena section; EP – endpoint; PSV – puncture of the subclavian vein; RT – rescue technique; V – visit.

A review of the previous literature, including our own randomised controlled trials, revealed a pneumothorax and haemothorax rate of about 3% for placement of a TIVAP by PSV; however, there is no risk if implantation by SCD is successful [14-18]. SCD was successful in 71 to 94% of cases (including the RT), therefore, about 17.5% of patients underwent PSV as a secondary implantation technique. Thus, the overall risk of pneumothorax and haemothorax for the open strategy is about 0.5% (0.175 × 0.03). Assuming a difference of 2.5%, a trial with a desired power of 0.9 and a type 1 error of 0.05 would need to include 577 patients per group to detect this difference in pneumothorax and haemothorax frequency when applying a chi-square test. No missing values for the primary endpoint are assumed, so a total of 1,154 patients have to be randomised into the two intervention groups. A Consolidated Standards of Reporting Trials (CONSORT) flow chart is provided in Figure 1.

### Eligibility

#### Inclusion criteria

Only adult patients (>18 years) with an oncological disease, scheduled for primary elective TIVAP implantation on the designated side, will be included. Furthermore, all subjects will have to be able to understand the character and individual consequences of this trial, and will have to provide written informed consent before inclusion.

#### Exclusion criteria

Patients who are participating in another interventional trial which could interfere with the primary endpoint of this trial will not be admitted.

#### Subject withdrawal criteria

PORTAS-3 trial participants will be withdrawn at their own request or if, in the investigator’s opinion, continuation of the trial would be detrimental to the subject’s wellbeing. Withdrawn patients will be included in the final report of the trial to ensure complete transparency.

### Informed consent

All patients scheduled for TIVAP implantation at one of the participating centres will be screened for eligibility and will be informed about the PORTAS-3 trial during a pretreatment visit. The patients will be asked to give their informed consent after all study procedures, potential risks and benefits and the data handling have been explained to them in detail.

### Randomisation and operations for minimising bias

#### Minimisation of systematic bias

Consecutively screened and eligible patients will be included at each centre after initiation of the study. In order to achieve comparable intervention groups for known and unknown risk factors, patients will be allocated randomly to the two treatment groups in balanced permuted blocks, and stratified by centre using the central web-based software “Randomizer”, provided by the Institute of Medical Informatics, Statistics and Documentation of the Medical University of Graz. To avoid any potential for predicting the group allocation of future patients, the block length is fixed in a separate document that is withheld from the study site. In addition, persons in charge of randomisation procedures as described above will not be able to read or edit the randomisation design chosen within the software.

#### Minimisation of treatment bias

Prior to study start, staff at all participating centres will be personally trained and introduced to all study-specific procedures during a collaborative investigator meeting. In addition, all procedures will be predefined in a study-specific monitoring manual. Participating centres will be updated on a regular basis to ensure comparable treatment of patients. All centres will use the same TIVAP device (Celsite® Epoxid Port, B Braun Medical, Boulogne-Billancourt, France). Antibiotic prophylaxis will be given to patients at risk of endocarditis according to the local standards and guidelines. In other cases, peri-operative prophylactic antibiotics can be administered at the discretion of the operating surgeon.

#### Minimisation of measurement bias

Blinding of patients and surgeons is not possible due to the nature of the surgical procedures to be evaluated. However, the chosen primary endpoint is not expected to be prone to measurement bias, as pneumothorax and haemothorax are defined exclusively and can be assessed objectively [19].

### Trial treatment

Since the PORTAS-3 trial is a pragmatic trial comparing two surgical strategies, only the key features of the compared trial treatments will be predefined by the protocol. Apart from these key features, the interventions can be performed according to the individual centre’s routine and the surgeon’s standard practice. The side for TIVAP implantation will depend on surgeon’s or patient’s preference. Factors that may influence the choice of implantation side include the following: breast cancer on one side, left- or right-handedness, left or right subclavian vein is occluded by a thrombosis, patient’s wish, previous insertion of port catheter and dialysis shunt on the left or right arm.

All patients will be placed in a 5° reverse Trendelenburg position. Each patient’s neck, chest and shoulders will be prepared and draped in the customary sterile manner. Antibiotic prophylaxis will be given to patients at risk of endocarditis according to the local standards and guidelines. In other cases, peri-operative prophylactic antibiotics can be administered upon the decision of the operating surgeon. A study nurse will be present to document the intervention. The intervention will be continued in compliance with the randomisation result.

#### Intervention group A (open strategy)

**Figure 2:**
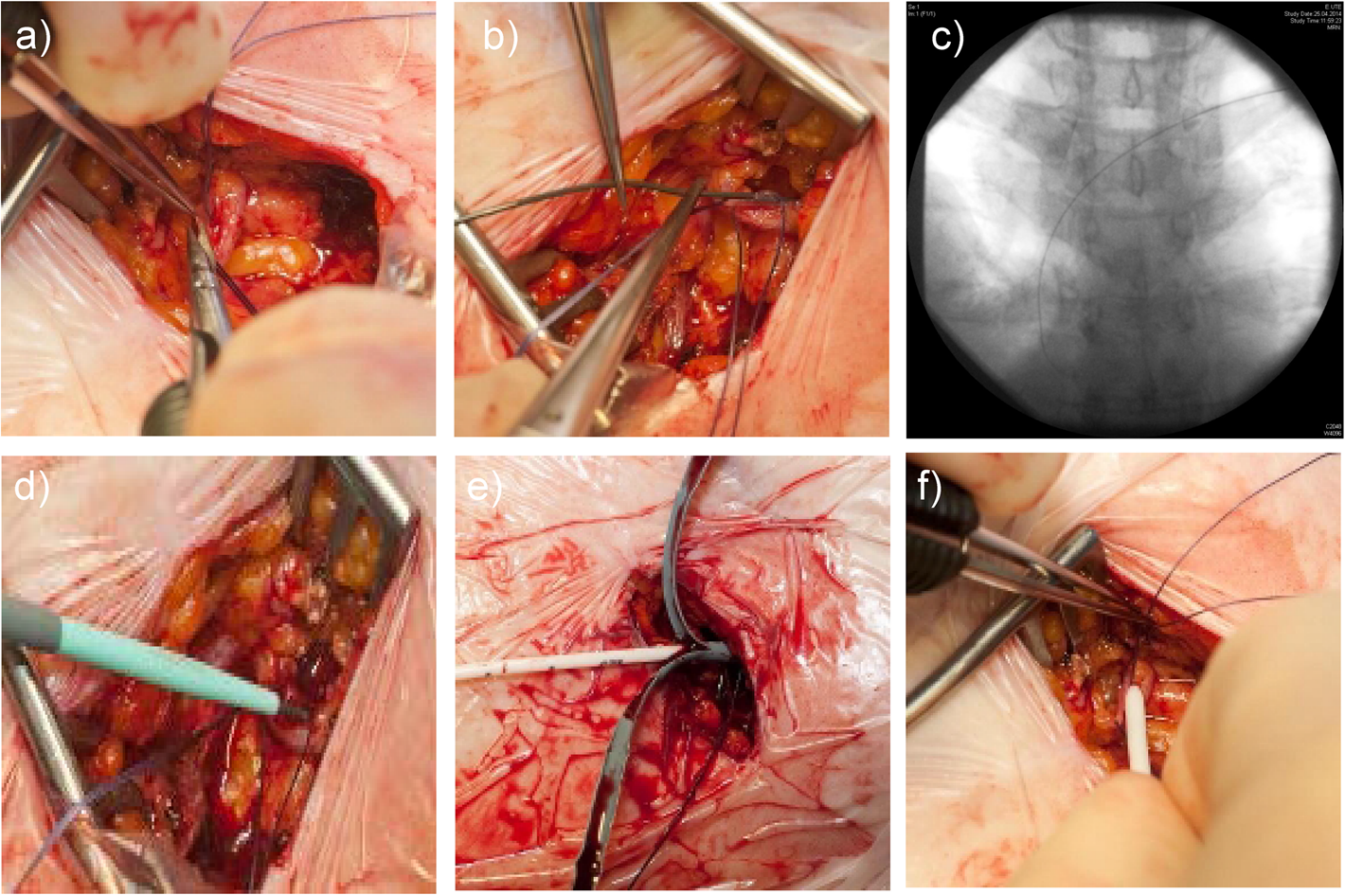
**Photo documentation of the rescue technique. a)** Ventral transection of cephalic vein. **b)** Introduction of a guide wire due to narrow cephalic vein. **c)** Radiograph of intravascular course of the guide wire. **d)** Introduction of vein dilator and peel-away sheath. **e)** Insertion of catheter and removal of peel-away sheath. **f)** Correctly placed catheter in cephalic vein and fixation.

Following the infiltration of local anaesthesia in sterile fashion into the skin and subcutaneous layer, a cutaneous incision will be made over the deltopectoral sulcus region to expose the cephalic vein. The cephalic vein will be ligated distally and encircled cranially with an resorbable suture. The vein will be transected ventrally (Figure 2a) and the catheter, flushed with heparinised saline, will be introduced. If simple SCD is not successful, the RT will be used as a second step. Therefore, catheter insertion will be supported by use of a guide wire (Figure 2b, c), vein dilator and peel-away sheath through the cephalic vein (Figure 2d). The guide wire and dilator will be removed, and the catheter easily introduced through the peel-away sheath, which can be removed afterwards (Figure 2e, f).

Correct positioning will be controlled via X-ray, fluoroscopy (tip of the catheter in the superior vena cava just at the level of bronchial bifurcation) or intraatrial electrocardiogram. The catheter will be connected to the port chamber. Using the same incision, a subcutaneous pocket will be prepared by blunt preparation on the pectoral fascia. The port chamber will be fixed on the fascia of the pectoral muscle with three single non-absorbable sutures. Wound closure will be performed in accordance with the local standards of the trial centres. Flow for blood withdrawal and infusion will be tested via cutaneous puncture (Huber needle). To complete the procedure, the system will be blocked by means of saline with or without heparin, according to the local standards.

#### Intervention group B (closed strategy)

Guidance for the puncture by means of sonography, fluoroscopy or the landmark technique is permitted. For fluoroscopy guidance, patients receive a peripheral venous catheter on the side of the planned puncture location to administer the contrast agent and check that the subclavian vein is not obstructed by a thrombosis (roadmap technique). For sonography guidance, a standard ultrasound device can be used to visualise the subclavian vein. The landmark technique uses anatomical landmarks (clavicle, jugular fossa, and so forth) to identify the right position for PSV. Local anaesthesia will be infiltrated in sterile fashion into the skin and periosteum of the clavicle. The location of the puncture will be marked between the proximal and medial third of the clavicle. A skin incision can be performed above the puncture location.

After that, the subclavian vein will be punctured using the Seldinger technique and a guide wire will be introduced. An introducer sheath will be passed over the guide wire into the vein. The guide wire will be removed and the port catheter introduced through the introducer sheath. Correct position will be checked by X-ray, fluoroscopy or intraatrial electrocardiogram. A subcutaneous pocket will be prepared by blunt preparation on the pectoral fascia through the same incision. Alternatively, a second skin incision can be performed and a subcutaneous pocket will then be prepared bluntly on the pectoral fascia. The port chamber will be fixed on the fascia of the pectoral muscle with three single non-absorbable sutures. Wound closure will be performed in accordance with the local standards of the trial centres. Correct position of the port catheter will be checked again through X-ray or fluoroscopy (tip of the catheter in the superior vena cava just at the level of bronchial bifurcation). Flow for blood withdrawal and infusion will be tested via cutaneous puncture (Huber needle). To complete the procedure the system will be blocked by means of saline, with or without heparin, according to local standards.

### Primary and secondary endpoints

#### Primary endpoint

The primary endpoint will be the frequency of post-interventional pneumothorax and haemothorax. Pneumothorax and haemothorax will be defined as the presence of air or blood in the pleural space, respectively. The rate of pneumothorax and haemothorax will be assessed two hours post-implantation by means of a chest X-ray (in inspiration) by the responsible physician if PSV was performed. In the event that SCD with or without RT is successful, a chest X-ray will be performed only in patients with suspicion or symptoms of pneumothorax or haemothorax (for example dyspnoea), since SCD has no associated risk of these conditions. A copy of the post-operative pneumothorax and/or haemothorax control radiograph will be saved in digital form, using radiological image viewing software (such as Centricity®, GE Healthcare, Buckinghamshire, United Kingdom), or a copy of the original radiograph will be archived. The presence of pneumothorax and haemothorax will be documented in the case report form (CRF). At follow-up visit three on day 30, the study nurse will also ask the patient about further post-operative occurrence of pneumothorax and haemothorax and check the patient’s documents for further diagnostic and treatment procedures in this regard.

#### Secondary endpoints

##### Duration of port implantation procedure

For the open strategy, the time from cutaneous incision to last knot of intracutaneous suture and the time from the patient entering the intervention room until the patient has left, will be measured. For the closed strategy, the time from first intervention on the patient (that is, first sonography, first radiography or first puncture) to the last knot of the intracutaneous suture, and the time from the patient entering the intervention room until the patient has left, will be measured. Both time measurements will be documented in the CRF on the day of intervention by the surgeon or a study nurse.

##### Subjective tolerability of intervention

This is defined as a subjective rating of the tolerability of the intervention by the patient. It will be assessed on the day of operation, after the intervention has taken place*.* Patients will be asked two questions rating the tolerability of the intervention on a standardised Likert scale (1 = well tolerable to 5 = intolerable). Furthermore, the willingness of the patients to repeat the intervention under the same conditions will be assessed. The answers will be documented in the CRF.

##### Primary success rate

Primary success is defined as correct positioning of the tip of the catheter in the superior vena cava with the randomised strategy at the intended side, controlled intra- or post-operatively by radiography, and correct function of the catheter, checked by blood sampling and infusion of fluid. Primary success will be assessed post-operatively by the responsible physician and documented in the CRF. A copy of the intra-operative radiograph showing the catheter in the correct position will be saved in digital form using radiological image viewing software (for example, Centricity), or a copy of the original radiograph will be archived.

##### Thirty-day mortality

This is defined as death during the first 30 days after the operation from causes related or unrelated to TIVAP implantation. In the event of not being able to talk to the patient at the 30-day telephone interview, the study nurse will contact the relatives or family physician to ask them whether the patient has died, and if so, the cause of death.

##### Thirty-day morbidity

This is defined as the occurrence of any of the complications listed in Table 1, known as possible peri- and post-operative complications of TIVAP implantation leading to deviation from the expected post-operative course. Peri- and post-operative complications of port implantation will be recorded with tick boxes in the CRF on the day of operation and at visit three (30 days after operation, by telephone questionnaire) by the surgeon and/or study nurses. All complications will be rated according to the Clavien-Dindo classification of surgical complications (Table 2) [20]. In the event of not being able to talk to the patient at the 30-day telephone interview, the study nurse will contact the relatives or family physician to ask them for information regarding the follow-up criteria.

**Table 1:** **Definitions of peri- and post-operative complications**

**Complication**	**Definition**
Intra-operative lesion of nerves	Clinical diagnosis and/or EMG findings
Dislocation of the catheter and/or the port chamber	Radiological finding
Incompatibility of contrast agent	Any allergic reaction against a contrast agent needing medical treatment with drugs
Thrombosis	Sonographic findings and/or phlebography
Post-operative bleeding	Clinical diagnosis during reoperation
Haematoma	Clinical diagnosis, no reoperation necessary
Disconnection or breakage of the catheter	Radiological findings, findings after explantation
Extravasation of injected fluid	Radiological findings and/or clinical diagnosis
Wound infection	Clinical diagnosis, wound has to be reopened and/or antibiotic treatment
Catheter sepsis	Two or more of the following symptoms:
	Temperature over 38.3°C or under 36°C;
	Heart rate over 90 beats per minute;
	Respiration rate over 20 breaths per minute, PaCO_2_ < 32 mmHg (spontaneous breathing) or PaO_2_/FiO_2_ < 200 mmHg (mechanical ventilation);
	Total peripheral WBC count >12 G/L or WBC <4.0 G/L or >10% immature neutrophils (bands), regardless of total peripheral WBC count;
	Plasma C-reactive protein >2 SD above normal value and positive findings in bacteriology of the port catheter pike
Cutaneous necrosis	Clinical diagnosis and/or histological finding
Pinch-off phenomenon	Radiological findings and/or clinical diagnosis

EMG: electromyography; PaCO2: partial pressure of carbon dioxide; PaO2: partial pressure of oxygen; FiO2: fraction of inspiratory oxygen; mmHg: millimetres of mercury; WBC: white blood cells.

**Table 2:** **Clavien**-**Dindo classification**

**Grade**	**Definition**
Grade I	Any deviation from the normal post-operative course without the need for pharmacological treatment or surgical, endoscopic or radiological interventions. Allowed therapeutic regimens are: drugs as antiemetics, antipyretics, analgesics, diuretics and electrolytes, and physiotherapy. This grade also includes wound infections opened at the bedside.
Grade II	Requiring pharmacological treatment with drugs other than those allowed for grade I complications. Blood transfusions and total parenteral nutrition are also included.
Grade III	Requiring surgical, endoscopic or radiological intervention.
Grade IIIa	Intervention not under general anaesthesia
Grade IIIb	Intervention under general anaesthesia
Grade IV	Life-threatening complication (including central nervous system complications)* requiring intensive care unit management
Grade IVa	Single-organ dysfunction (including dialysis)
Grade IVb	Multi-organ dysfunction
Grade V	Death
Suffix ‘d’	If the patient is suffering from a complication at the time of discharge, the suffix ‘d’ (for ‘disability’) is added to the grade. This label indicates the need for follow-up to fully evaluate the complication.

*including brain hemorrhage, ischemic stroke, subarachnoidal bleeding, but excluding transient ischemic attacks.

##### Dose rate of radiation

This is defined as the product of dose rate and area of radiation (cGy × cm^2^). The correctness of documentation will be verified by a study nurse. The exact value will be documented in the CRF on the day of intervention.

##### Skill of surgeons

Surgeons will be classified in the CRF as follows: 1 to 10, 11 to 25, 26 to 50 or >50 TIVAP implantations. Whether or not surgeons have a board certificate on the day of intervention will be documented. It will also be documented whether another experienced surgeon was called in, and instances of complex or unsuccessful primary implantation will be recorded.

### Description of trial visits

The specific data to be collected at the trial visits are presented in Table 3.

**Table 3:** **Data to be collected at trial visits one to three**

**Visit**	**1**	**2**	**3**
	**Screening**	**Day of operation**	**Post-operative day 30**
Inclusion and exclusion criteria, and written informed consent	**X**		
Baseline demographic data	**X**		
Randomisation		**X**	
Primary endpoint		**X**	**X**
Tolerability of intervention		**X**	**X**
Duration of procedure		**X**	
Primary success rate		**X**	
Survival			**X**
Complications		**X**	**X**
Serious adverse events		**X**	**X**

#### Visit one

If a subject is included, the following baseline data will be recorded in the CRF: gender, age (years), height (cm), weight (kg), relevant comorbidities (chronic obstructive pulmonary disease, lung emphysema or asthma), underlying malignant disease necessitating TIVAP, current antibiotic treatment, current CHT, smoking status, alcohol abuse, Karnofsky score.

#### Visit two

Prior to the surgical intervention, patients will be randomised to either the open or closed strategy, and the treatment assignment will be documented. Furthermore, the following details will be documented: performed intervention (SCD or PSV), surgeon’s expertise, duration of procedure, site of TIVAP implantation, primary success rate, rate of pneumothorax and haemothorax (primary endpoint), serious adverse events (SAE), study port implanted (yes or no), peri-operative prophylactic antibiotics given (yes or no), exact documentation of the executed procedure and rating of tolerability of intervention.

#### Visit three

Patients will be called by a study nurse on day 30 post-operation and asked about SAE and primary and secondary endpoints by means of a standardised questionnaire. All data will be documented in the CRF.

### Safety aspects

In the PORTAS-3 trial only SAEs meeting one of the following criteria will be documented: resulting in death, is immediately life-threatening, requiring or prolonging hospitalisation, resulting in persistent or significant disability or incapacity and other substantial medical reasons.

From the day the patient has been randomised until the regular end of trial at 30 days follow-up, or until premature withdrawal of the patient, all SAE will be documented on an SAE form, available in the investigator site file. Assessment of SAE will be based on surgical findings and the clinical course of the patient, and will be carried out by the investigators at the participating trial centres. They will be furnished with written recommendations on how to assess the causal connection with trial interventions. An SAE will have to be reported to the principal investigator by the attending physician within 24 hours after it becomes known.

#### Study-specific serious adverse events

As it can be anticipated that many events fulfilling the SAE criteria will be caused by the underlying disease or treatment and will not be related to trial intervention (for example, hospitalisation due to neutropenic fever after CHT), only SAEs not related to the underlying disease or treatment will have to be reported. Anticipated SAEs due to the underlying disease that do not have to be reported include the following:

1. hospitalisation due to progress of underlying disease;
2. hospitalisation due to bone marrow toxicity of CHT, including:
  - neutropenic fever,
  - anaemia requiring transfusion of red blood cell concentrates,
  - haemorrhagic events necessitating intervention/transfusion due to thrombocytopenia;
3. hospitalisation due to severe nausea, vomiting and exsiccosis caused by CHT;
4. hospitalisation due to severe impairment of hepatic/renal function related to CHT.

The occurrence of pneumothorax and haemothorax will be documented as SAEs for safety reasons.

### Analysis

#### Hypotheses

The primary endpoint is the occurrence of pneumothorax and haemothorax after implantation of a TIVAP. To formalise the statistical approach, the following notation will be used: p_open_/p_closed_, that is, rate of occurrence of pneumothorax and haemothorax after implantation of a TIVAP in the open-strategy group (open)/closed-strategy group (closed).

The following two-sided test problem is defined:

$$ {\mathrm{H}}_0:{\mathrm{p}}_{\mathrm{open}}={\mathrm{p}}_{\mathrm{closed}}\mathrm{versus}\ {\mathrm{H}}_1:{\mathrm{p}}_{\mathrm{open}}\ne {\mathrm{p}}_{\mathrm{closed}} $$

#### Analysis sets

Each patient’s allocation to the different analysis populations (full analysis set (FAS) according to the intention-to-treat (ITT) principle, per protocol (PP) analysis set and safety analysis set) will be defined prior to the analysis. The allocation will be documented in the statistical analysis plan. During the data review, deviations from the protocol will be assessed as ‘minor’ or ‘major’. Major deviations from the protocol will lead to the exclusion of a patient from the PP analysis set.

#### Testing the primary hypothesis

The null hypothesis H0 will be assessed by testing the intervention effect in a logistic regression model that takes into account the covariates ‘intervention’ (open or closed strategy), ‘skill of surgeon’ (board certificate yes or no, number of TIVAP implanted), ‘body mass index’ (kg/m^2^), ‘Karnofsky index’ (percentage) ‘gender’ (female or male) and ‘age’ (years). A two-sided type I error rate of α = 0.05 will be applied. Confirmatory analysis will be primarily based on the FAS, which is consistent with the ITT principle by including all patients randomised to the two groups. This approach reflects the idea that the study should match the conditions in clinical practice as closely as possible.

#### Missing data

As explained above, it can be assumed that there will be no missing values with respect to the primary endpoint. If missing values occur, they will be replaced by means of the ICA-r method described by Higgins *et al*. [21].

#### Further analyses

In addition to the evaluation of the FAS a PP analysis will be performed including all randomised patients without major protocol violations. The secondary variables will be analysed descriptively by tabulation of the measures of the empirical distributions. According to the scale level of the variables, means, standard deviations, medians and first and third quartiles, as well as minimum and maximum or absolute and relative frequencies will be reported. Descriptive *P* values of the corresponding statistical tests comparing the treatment groups and associated 95% confidence intervals will be given.

The homogeneity of the treatment groups will be described by comparison of the demographic data and the baseline values. Statistical methods will be used to assess data quality and the homogeneity of the intervention groups. A safety analysis will be conducted and will include calculation and comparison of frequencies and rates of SAEs. All analyses will be done using SAS version 9.1 or higher (SAS Institute Inc., Cary, North Carolina, USA).

### Participating centres

The following centres are planned to participate in the PORTAS-3 trial: Ansbach Hospital, Berlin Park Hospital Weißensee, Municipal Hospital Braunschweig, University Hospital Dresden, Evangelical Hospital Gelsenkirchen, Eichert Hospital/Göppingen Rural District Hospitals, University Hospital Heidelberg, Salem Hospital Heidelberg, Heidenheim Hospital, Ingolstadt Hospital, Memmingen Hospital, Lukas Hospital Neuss, Esslingen District Hospitals/Nürtingen Hospital, Passau Hospital, University Hospital Regensburg, “Gesundheitszentren Rhein-Neckar” Hospital Sinsheim and Joseph Hospital Warendorf.

### Trial organisation

All patients scheduled for TIVAP implantation at one of the participating centres will be screened for eligibility by the respective study centre, and the results will be documented in a screening log. The participating centres perform approximately 3,882 TIVAP implantations per year (estimated from preliminary screening over a two-month period). Therefore, a recruitment period of 18 months is expected to be sufficient to randomise 1,154 patients. Independent data management and statistical analyses will be performed by the Institute of Medical Biometry and Informatics of the University of Heidelberg, in compliance with a pre-specified statistical analysis plan.

### Ethical and legal aspects

All procedures set out in the protocol pertaining to the conduct, evaluation and documentation of the PORTAS-3 trial are designed to ensure that all persons involved in the trial abide by the principles of Good Clinical Practice [22] and the ethical principles described in the current revision of the Declaration of Helsinki [23]. The trial will be carried out in accordance with local legal and regulatory requirements (such as the German Federal Data Protection Act, 1990 [last change 25 February 2015]).

The protocol has already been approved by the independent ethics committee (IEC) of the medical faculty of the University of Heidelberg, and secondary approval of the corresponding ethical bodies of all other participating centres has been obtained. A list containing the names and reference numbers of all ethical bodies is provided as an additional file (Additional file 1). Any amendments will be submitted to all of the IEC. The IEC will also be informed of the end of the trial. The trial protocol has been issued in accordance with the recommendations of the CONSORT and Standard Protocol Items: Recommendations for Interventional Trials (SPIRIT) guidelines (Additional file 2) [24,25].

## Discussion

TIVAP implantation is one of the most common general surgical procedures [3] and is performed routinely by either SCD or PSV. However, only few randomised controlled trials [4,6,9,17] and no multicentre trials compared these techniques for their potential risks and benefits. The aim of the PORTAS-3 trial is to compare a primary open strategy (SCD with RT if necessary; PSV if the aforementioned fail) to a primary closed strategy (PSV; SCD if PSV fails) in regard to the frequency of post-operative pneumothorax and haemothorax, with the hypothesis that the open strategy is able to significantly reduce the risk of pneumothorax and haemothorax occurrence.

The PORTAS-3 trial is designed as a pragmatic trial, since the objective is to depict the real medical treatment situation. Therefore, just some key steps of the interventions are predefined, and the rest can be performed according to local practice of the centres. For instance, in PSV either sonography, fluoroscopy or anatomical landmarks can be used for guidance of the puncture. Some authors suggested that sonographic guidance should be the method of choice for vascular cannulation due to improved safety [26]. Even though ultrasound is widely available nowadays, this still does not represent comprehensive clinical practice in a lot of hospitals, and a recent Cochrane review on ultrasound guidance for subclavian vein catheterization [27] could only show a reduced risk of arterial cannulation, and no reduction for other complications like pneumothorax and haemothorax.

The results of the PORTAS-3 trial will be applicable to a wide range of patients and could influence future guidelines and decision-making. Healthcare costs might be reduced by the reduction of complications and necessary treatment and hospitalization for these complications. Furthermore, required treatment of patients (for example, with CHT) will not be delayed by potential complications.

## Trial status

The PORTAS-3 trial has already started recruitment of patients in November 2014 and recruitment is currently ongoing and is expected to be complete in the first quarter of 2016.

## Additional files

Additional file 1:
**Ethical approvals: List of participating centres with corresponding ethics committees and reference numbers of the ethical votes.**
Additional file 2:
**SPIRIT checklist: SPIRIT checklist with references for each point of the checklist to the page of the protocol publication, on which this point is addressed.**

## Abbreviations

CHT: chemotherapy
CRF: Case report form
FAS: Full analysis set
IEC: Independent ethics committee
ITT: Intention-to-treat
PP: Per protocol
PSV: Puncture of the subclavian vein
RT: Rescue technique
SAE: Serious adverse event
SCD: Surgical cut-down of the cephalic vein
SDGC: Study Center of the German Surgical Society
TIVAP: Totally implantable venous access port
