# Supplementary material for: Frequency of pneumothorax and haemothorax after primary open versus closed implantation strategies for insertion of a totally implantable venous access port in oncological patients: study protocol for a randomised controlled trial
Source: Trials. 2015 Mar 31;16:128. doi: 10.1186/s13063-015-0643-z (PMC4396913; doi:10.1186/s13063-015-0643-z)
Supplement: Additional file 1: — Ethical approvals: List of participating centres with corresponding ethics committees and reference numbers of the ethical votes. [file 13063_2015_643_MOESM1_ESM.docx]

|  | **Centre** | **Ethics committee** | **Reference number** |
| --- | --- | --- | --- |
| 1 | **Ansbach Hospital** **Department of Surgery I General and Visceral Surgery, surgical oncology**  ANregiomed Klinikum Ansbach Chirurgische Klinik I  Allgemein- und Viszeralchirurgie, chirurgische Onkologie | **Ethics Committee of the Bavarian State Chamber of Physicians**  Ethikkommission der bayerischen Landesärztekammer | Mb BO 14040 |
| 2 | **Berlin Park Hospital Weißensee General, Visceral and minimal invasive Surgery**  Park-Klinik Weißensee (Berlin)  Allgemein-, Viszeral- und minimal-invasive Chirurgie | No approval required |  |
| 3 | **Municipal Hospital Braunschweig Department of General and Visceral Surgery**  Städtisches Klinikum Braunschweig Klinik für Allgemein- und Viszeralchirurgie | **Medical Association Lower Saxony  Ethics committee**  Ärztekammer Niedersachsen Ethikkommission | Ar/231/2014 |
| 4 | **University hospital Carl Gustav Carus Dresden Department for Visceral, Thoracic and Vascular Surgery**  Universitätsklinikum Carl Gustav Carus Dresden Klinik und Poliklinik für Viszeral-, Thorax-  und Gefäßchirurgie | **Technical University Dresden** **Ethics committee**  Technische Universität Dresden Ethikkommission | EK 344092014 |
| 5 | **Evangelical Hospital Gelsenkirchen Department of General, Visceral and Vascular Surgery**  Evangelische Kliniken Gelsenkirchen  Klinik für Allgemein-, Viszeral- und Gefäßchirurgie | **Ethics committee of the Medical Association Westphalia Lippe and the medical faculty of the Westphalian Wilhelms-University**  Ethikkommission der Ärztekammer Westfalen Lippe und der medizinischen Fakultät der Westfälischen Wilhelms-Universität | 2014-520-b-S |
| 6 | **Eichert Hospital/ Göppingen Rural District Hospitals Department of Surgery Visceral and Thoracic Surgery**  ALB FILS KLINIKEN (Göppingen) Klinik am Eichert Allgemeinchirurgische Klinik Schwerpunkt Viszeral- und Thoraxchirurgie | **State Chamber of Physicians Baden-Wuerttemberg Ethics committee**  Landesärztekammer Baden-Württemberg Ethikkommission | B-F-2014-075 |
| 7 | **Department of General, Visceral and Transplantation Surgery**  **University Hospital Heidelberg**  Klinik für Allgemein-, Viszeral- und Transplantationschirurgie  Universitätsklinikum Heidelberg | **Ethics committee of the medical faculty of the University of Heidelberg**  Ethikkommission  der Medizinischen Fakultät der  Ruprecht-Karl-Universität Heidelberg | S-308/2014 |
| 8 | **Salem Hospital Heidelberg** **Department of Surgery** Krankenhaus Salem  Chirurgische Abteilung | **Ethics committee of the medical faculty of the University of Heidelberg**  Ethikkommission der Medizinischen Fakultät der Ruprecht-Karl-Universität Heidelberg | S-308/2014 |
| 9 | **Heidenheim Hospital Visceral, Thoracic and Vascular Surgery**  Klinikum Heidenheim  Viszeral-, Thorax- und Gefäßchirurgie | **State Chamber of Physicians Baden-Wuerttemberg**  Landesärztekammer Baden-Württemberg  Ethikkommission | B-F-2014-075 |
| 10 | **Ingolstadt Hospital Department of Surgery I**  Klinikum Ingolstadt  Chirurgische Klinik I Allgemein-, Viszeral-, Thorax- und Endokrine Chirurgie | **Ethics Committee of the Bavarian State Chamber of Physicians**  Ethikkommission der bayerischen Landesärztekammer | Mb BO 14040 |
| 11 | **Memmingen Hospital Department for General, Visceral, Vascular and Thoracic Surgery**  Klinikum Memmingen  Klinik für Allgemein-, Visceral-, Gefäß- und Thoraxchirurgie | **Ethics Committee of the Bavarian State Chamber of Physicians**  Ethikkommission der bayerischen Landesärztekammer | Mb BO 14040 |
| 12 | **Lukas Hospital Neuss Department of General, Visceral, Thoracic and Vascular Surgery**  Lukaskrankenhaus Neuss  Klinik für Allgemein-, Viszeral-, Thorax- und Gefäß-Chirurgie | **Medical Association North-Rhine**    Ärztekammer Nordrhein Ethikkommission | 2014389 |
| 13 | **Esslingen District Hospitals / Nürtingen Hospital Department of General and Visceral Surgery**  Kreiskliniken Esslingen Klinik Nürtingen Klinik für Allgemein- und Viszeralchirurgie | **State Chamber of Physicians Baden-Wuerttemberg**  Landesärztekammer Baden-Württemberg  Ethikkommission | B-F-2014-075 |
| 14 | **Passau Hospital Department of Surgery**  Klinikum Passau  Chirurgische Klinik | **Ethics Committee of the Bavarian State Chamber of Physicians**  Ethikkommission der bayerischen Landesärztekammer | Mb BO 14040 |
| 15 | **University Hospital Regensburg Department of Surgery**  Universitätsklinikum Regensburg  Klinik und Poliklinik für Chirurgie | **Ethics Committee of the Bavarian State Chamber of Physicians**  Ethikkommission der bayerischen Landesärztekammer | Mb BO 14040 |
| 16 | **GRN Hospital Sinsheim Department of General and Visceral Surgery**  GRN-Klinik Sinsheim  Allgemein- und Viszeralchirurgie | **Ethics committee of the medical faculty of the University of Heidelberg**  Ethikkommission der Medizinischen Fakultät der Ruprecht-Karl-Universität Heidelberg | S-308/2014 |
| 17 | **Joseph Hospital Warendorf Department of General, Visceral and Vascular Surgery**  Josephs-Krankenhaus Warendorf Allgemein-, Viszeral- und Gefäßchirurgie | **Ethics committee of the Medical Association Westphalia Lippe and the medical faculty of the Westphalian Wilhelms-University**  Ethikkommission der Ärztekammer Westfalen Lippe und der medizinischen Fakultät der Westfälischen Wilhelms-Universität | 2014-520-b-S |
